# Design and Evaluation of a Novel Venturi-Based Spirometer for Home Respiratory Monitoring

**DOI:** 10.3390/s24175622

**Published:** 2024-08-30

**Authors:** Mariana Ferreira Nunes, Hugo Plácido da Silva, Liliana Raposo, Fátima Rodrigues

**Affiliations:** 1 Department of Bioengineering, Instituto Superior Técnico, University of Lisbon, 1949-001 Lisbon, Portugal; hsilva@lx.it.pt; 2 IT—Instituto de Telecomunicações, Instituto Superior Técnico, 1949-001 Lisbon, Portugal; 3 LUMLIS—Lisbon Unit for Learning and Intelligent Systems, 1049-001 Lisbon, Portugal; 4 Pulmonology Department, Santa Maria Local Health Unit, 1769-001 Lisbon, Portugal; lraposo@esscvp.eu (L.R.); fatima.pneumo@gmail.com (F.R.); 5 Cardiopneumology Teaching Department, Higher School of Health of The Portuguese Red Cross, 1300-125 Lisbon, Portugal; 6 Institute of Environmnetal Health, Lisbon Medical School, Lisbon University, 1649-026 Lisbon, Portugal

**Keywords:** spirometry, respiration monitoring, pressure sensor, Bernoulli principle, Venturi tube

## Abstract

The high cost and limited availability of home spirometers pose a significant barrier to effective respiratory disease management and monitoring. To address this challenge, this paper introduces a novel Venturi-based spirometer designed for home use, leveraging the Bernoulli principle. The device features a 3D-printed Venturi tube that narrows to create a pressure differential, which is measured by a differential pressure sensor and converted into airflow rate. The airflow is then integrated over time to calculate parameters such as the Forced Vital Capacity (FVC) and Forced Expiratory Volume in one second (FEV_1_). The system also includes a bacterial filter for hygienic use and a circuit board for data acquisition and streaming. Evaluation with eight healthy individuals demonstrated excellent test-retest reliability, with intraclass correlation coefficients (ICCs) of 0.955 for FVC and 0.853 for FEV_1_. Furthermore, when compared to standard Pulmonary Function Test (PFT) equipment, the spirometer exhibited strong correlation, with Pearson correlation coefficients of 0.992 for FVC and 0.968 for FEV_1_, and high reliability, with ICCs of 0.987 for FVC and 0.907 for FEV_1_. These findings suggest that the Venturi-based spirometer could significantly enhance access to spirometry at home. However, further large-scale validation and reliability studies are necessary to confirm its efficacy and reliability for widespread use.

## 1. Introduction

Spirometry is regarded as the primary diagnostic tool for assessing respiratory diseases, playing a crucial role in diagnosing and monitoring both obstructive and restrictive lung conditions [1,2]. It evaluates lung function by measuring the maximum volume of air that can be forcefully exhaled after a deep inspiration, along with the corresponding airflow rate during this exhalation [3].

Traditionally, spirometry is conducted in clinical settings, with spirometers typically being desktop devices or large cabinet-sized machines operated by trained technicians [4]. However, scheduling such tests usually requires a doctor’s referral, thereby restricting access to spirometry. Delays or barriers in accessing care may hinder early diagnosis of respiratory diseases and limit opportunities for monitoring disease progression and treatment response [5]. As a result, there is a growing interest in home handheld spirometry [5].

The size, technical complexity, and the need for trained technicians of hospital-grade spirometers render them impractical for home settings. Therefore, the ongoing trend is toward smaller and more cost-effective spirometer devices that empower patients to perform spirometry tests independently as part of their daily routines [6]. However, existing portable spirometers still face significant limitations that hinder their effectiveness in non-clinical environments.

For instance, turbine flow meters, which rely on rotating blades to measure airflow, are affected by inertia. This can cause the blades to keep rotating even after exhalation has ceased, resulting in inaccurate flow measurements. Pneumotachographs, while commonly used in clinical settings, are prone to clogging from dirt or mucus, which can obstruct the airflow and affect measurement accuracy. Additionally, they are sensitive to the temperature, humidity, and atmospheric pressure of the surrounding air, necessitating frequent calibration.

In response to these limitations, Venturi-based spirometers emerge as a promising alternative [7,8,9]. By harnessing the Venturi effect—a principle where a constricted tube creates a pressure drop proportional to the flow rate—these devices simplify their design by eliminating moving parts and complex resistive elements. This design results in enhanced performance stability and reduces the necessity for frequent recalibration. As a result, Venturi-based spirometers offer consistent measurements with minimal maintenance needs, making them a more robust and reliable choice.

Due to its potential, this paper proposes the design and development of a spirometer prototype based on a Venturi tube and a differential pressure sensor. It is essential to clarify that this study is exploratory in nature. As a result, only one pressure sensor will be employed in this initial phase, meaning that the spirometer will exclusively measure expiratory flow rates. By leveraging the simplicity and effectiveness of the Venturi tube, this innovative approach aims to overcome current barriers and improve accessibility to accurate lung function assessment outside traditional clinical environments.

The remainder of this paper is organized as follows: Section 2 discusses the two types of spirometers, volume-sensing and flow-sensing, along with their respective advantages and disadvantages. Section 3 outlines the proposed approach, detailing the working principle, the design of the Venturi tube, and a comprehensive description of each component of the prototype. Section 4 covers the fluid flow analysis. Section 5 describes the laboratory evaluation process, including the calibration of the pressure sensor and calibration verification using a 3 L syringe. Section 6 presents the evaluation of the prototype with users in a hospital setting, comparing it to standard equipment, while Section 7 reports the results obtained from this evaluation. Finally, Section 8 presents the main conclusions derived from the study, and Section 9 discusses future work and next steps.

## 2. Background

Spirometers are generally classified as either volume-sensing or flow-sensing devices. Volume-sensing spirometers establish a closed system between the patient and the device, where changes in the individual’s lung volume correspond directly to changes in the volume within the spirometer container [10]. This change in volume is accurately measured by tracking the displacement of a bellows, piston, or bell [11]. Despite being highly accurate, these spirometers are characterized by multiple moving parts, making them challenging to clean and disinfect. Additionally, their design necessitates a container larger than the volume of air exhaled, contributing to their bulkiness [11,12,13]. Driven by the inherent limitations of volume-sensing spirometers, and propelled by advancements in electronic technology and software, the development of flow-sensing spirometers ensued.

Flow-sensing spirometers directly measure flow and can be further classified into various types based on the physical principle they employ as follows: pneumotachographs, turbine flow meters, heated-wire anemometers, and ultrasonic spirometers.

**Figure 1:**
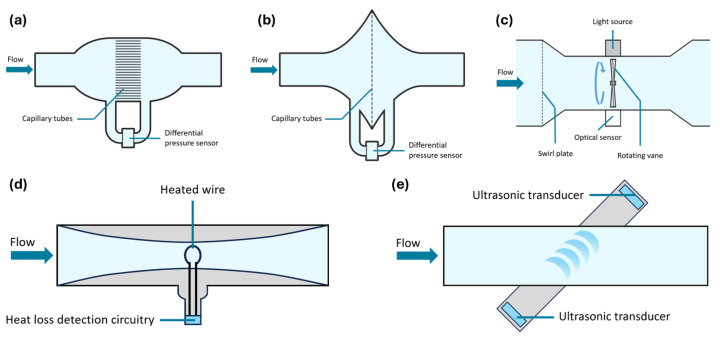
Types of flow-sensing spirometers. (**a**) Fleisch-type pneumotachograph. (**b**) Lilly-type pneumotachograph. (**c**) Turbine flow meter. (**d**) Heated-wire anemometer. (**e**) Ultrasonic spirometer. Adapted from [14,17].

The pneumotachograph consists of a tube with a resistive element, which can either be a set of narrow capillary tubes arranged parallel to the airflow (Fleisch type) or a fine wire mesh screen (Lilly type) [12,14], as depicted in Figure 1a and Figure 1b, respectively. While allowing air to flow through the tube, the resistive element causes a pressure drop. Under conditions of laminar flow, this pressure drop is directly proportional to the flow rate [14,15,16].

A turbine flow meter, as illustrated in Figure 1c, comprises a tube equipped with a swirl plate and a low-inertia vane [17]. When air passes through the tube, the swirl plate generates a vortex that causes the vane to rotate [17]. With each rotation, the vane interrupts an infrared light beam aimed at an infrared detector, generating an electrical impulse upon each interruption [12]. The frequency of these pulses corresponds to the flow rate of the air passing through the meter [12].

A heated-wire anemometer operates by heating a wire to a constant temperature at the center of a tube, as depicted in Figure 1d [14]. As air passes through the tube, it cools the heated wire, requiring an increase in electrical current to maintain the wire at the set temperature [12,14]. The amount of current required to sustain this temperature is directly proportional to the airflow rate [14].

The ultrasonic spirometer operates on the principle of the Doppler effect. As depicted in Figure 1e, two diagonally opposing piezoelectric transducers emit and receive ultrasonic waves in alternating directions [14]. When air flows through the tube, the ultrasonic pulse traveling in the direction of flow accelerates and reaches the opposing transducer more quickly [12,14]. Conversely, the ultrasonic pulse moving against the flow decelerates, requiring more time to reach the opposite transducer [12,14]. The difference in travel times between these pulses correlates directly with the airflow rate [12].

Flow-sensing spirometers have largely replaced volume-sensing spirometers due to the several advantages they offer over their traditional counterparts. These devices are more compact and lightweight, which renders them well-suited for portable systems [12]; they can also be easily integrated into a variety of complex testing systems, such as a body plethysmograph. Additionally, they require minimal maintenance and are easier to clean. Nevertheless, they still present limitations. Turbine flow meters, for instance, have several moving parts and are slow to respond to flow changes because of inertia. Hot-wire anemometers are unidirectional and sensitive to gas composition and temperature, and the wire is relatively fragile [14]. Meanwhile, the primary downside of ultrasonic spirometers is their high cost, limiting their widespread adoption [12].

Nowadays, the majority of clinical-grade spirometers are pneumotachographs. The main inconvenience of both Fleisch and Lilly pneumotachographs is their sensitivity to temperature, humidity, and atmospheric pressure, necessitating frequent calibration—at least daily and after each displacement. Pneumotachographs are also prone to clogging, which can alter their resistance and lead to inaccurate measurements.

The proposed Venturi-based spirometer operates similarly to pneumotachographs, employing a tube with a resistive element to create a pressure drop. However, instead of narrow capillary tubes or fine wire mesh screens, the pressure reduction in the Venturi tube occurs due to a narrowing of the tube itself. This simpler and more economical fabrication process eliminates the vulnerability to moisture and debris that affects traditional pneumotachographs.

## 3. Proposed Approach

### 3.1. Working Principle

The proposed spirometer employs the Venturi effect to achieve accurate airflow measurement, a direct result of Bernoulli’s principle. According to this principle, in an ideal fluid, the sum of the pressure $\left(p\right)$, kinetic energy $\left(\frac{1}{2}\rho v^{2}\right)$, and potential energy $\left(\rho gh\right)$ per unit volume remains constant at all points [18]. This is expressed in Equation (1), where ρ is the fluid density, $v_{i}$ is the average fluid speed in position *i*, *g* is the acceleration due to gravity, and $h_{i}$ is the height of point *i* in respect to the sea [18].

(1) $$p_{1}+\frac{1}{2}\rho v_{1}^{2}+\rho gh_{1}=p_{2}+\frac{1}{2}\rho v_{2}^{2}+\rho gh_{2}$$

When considering a horizontal tube, the acceleration of the fluid due to gravity can be disregarded. Additionally, the density and height of the fluid in the tube are assumed to be constant. Thus, Equation (1) can be simplified to Equation (2); under these conditions, the volumetric flow rate ($q_{v}$) is also constant, as per the continuity principle. This is expressed by Equation (3), where $A_{i}$ is the cross-sectional area at position *i* [7].

(2) $$p_{1}+\frac{1}{2}\rho v_{1}^{2}=p_{2}+\frac{1}{2}\rho v_{2}^{2}$$

(3) $$q_{v}=v_{1}\cdot A_{1}=v_{2}\cdot A_{2}$$

**Figure 2:**
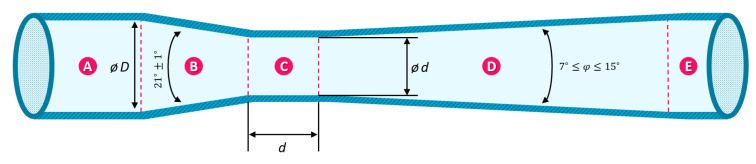
A cross-sectional view of a Venturi tube, showcasing the inlet section (A), the converging section (B), the constricted throat (C), the diverging section (D), and the outlet section (E).

A Venturi tube is a horizontal tube with a constricted region, as depicted in Figure 2. When fluid flows through the narrow section, it accelerates and experiences a decrease in pressure. By measuring the pressure difference between the tube’s entrance (section A in Figure 2, corresponding to position 1 in the equations) and the constricted throat (section C in Figure 2, corresponding to position 2 in the equations), the flow rate can be determined, as described by Equation (4). To obtain the volume of air passing through the Venturi tube, the flow rate can be integrated over time.

(4) $$q_{v}=C_{d}\cdot A_{2}\cdot \sqrt{\frac{2\cdot (p_{1}-p_{2})}{\rho \cdot \left[1-{\left(\frac{A_{2}}{A_{1}}\right)}^{2}\right]}}$$

In Equation (4), $C_{d}$ represents the coefficient of discharge, defined as the ratio of the actual flow rate through the Venturi tube to the theoretical flow rate [19]. This coefficient accounts for frictional losses, which are present in real-world applications and cause the actual flow rate to be less than the theoretical flow rate [19].

### 3.2. Venturi Tube Design

With the theoretical foundation established through Bernoulli’s principle and the Venturi effect, the next step is to translate these principles into a practical design for the Venturi tube used in the spirometer. The goal is to create a design that ensures precise and reliable measurement of airflow rates, which is essential for accurate respiratory function assessment. Achieving this requires careful consideration of various design parameters and choices.

One of the most critical aspects of the design is determining the optimal inlet and throat diameters, as these significantly influence the range of flow rates that the spirometer can accurately measure. The inlet diameter was established at 31 mm to ensure a secure fit for the filter/mouthpiece, as the standard diameter for these components is 30 mm [20,21]. The throat diameter was chosen to be 8 mm, which allows for the measurement of flow rates ranging from 0 to 12 L/s, assuming that a pressure differential of up to 5 pounds per square inch (PSI) (approximately ±352 cm H_2_O) can be accurately measured. For future iterations, a greater throat diameter should be considered, as a spirometer needs to measure flows in the range of 0 to 14 L/s [14,22]. However, it is important to note that increasing the throat diameter may reduce accuracy at lower flow rates.

The remaining geometrical parameters of the Venturi tube were carefully chosen to adhere to the ISO-5167-4:2003 standard [23]. In particular, the converging section’s length was fixed at 62 mm, corresponding to a converging angle of approximately 21.02°, while the diverging section’s length was specified at 88 mm, resulting in an angle of approximately 14.89°.

**Figure 3:**
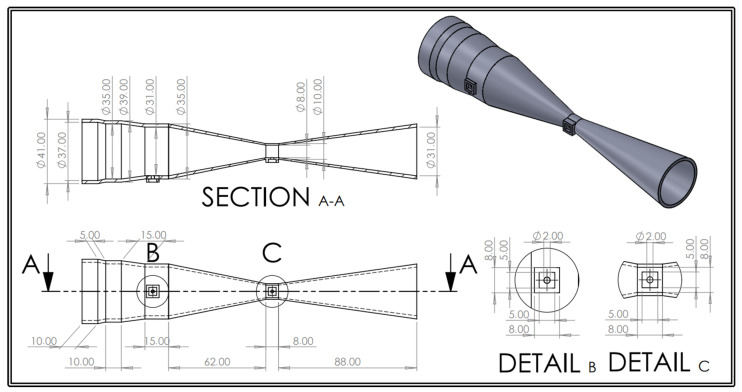
Technical drawing of the revised Venturi tube; all dimensions are presented in millimeters. Detail B and Detail C are proportionally scaled at a 1:2 ratio relative to the main drawing.

**Figure 4:**
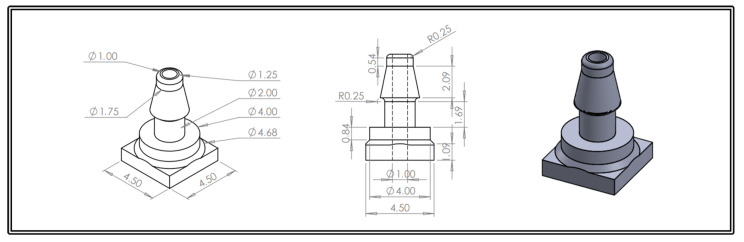
Technical drawing of the pressure tap; all dimensions are presented in millimeters.

The final design of the Venturi tube, including the adjustments made to accommodate the bacterial filter and calibration syringe, is depicted in Figure 3. The design also includes two openings for pressure taps, which are shown separately in Figure 4. For more detailed information on the design evolution and manufacturing process, please refer to Appendix A.

### 3.3. Hardware Components

Besides the Venturi tube, the proposed spirometer prototype incorporates several components crucial for its functionality. These include a differential pressure sensor to measure pressure differences between the Venturi tube’s inlet and throat, a mouthpiece with an integrated bacterial filter to ensure hygiene standards, a circuit board for data acquisition and wireless streaming of pressure sensor data, and silicone tubing for establishing airtight connections between the components.

When selecting the appropriate pressure sensor, priority was given to its differential nature and compatibility with human respiratory dynamics. After evaluating available options, the Honeywell SSCMRRN005PDAA3 pressure sensor was chosen [24]. This differential type sensor offers a pressure range of ±5 PSI (≈±352 cm H_2_O) [24]. This range was deemed sufficient because the European Respiratory Society (ERS) statement on respiratory muscle testing recommends a pressure sensor range of ±300 cm H_2_O, making it reasonable to assume that the maximum respiratory pressure in humans is below this value [25]. Nevertheless, since the spirometer measures pressure differences rather than absolute pressures, a smaller range would potentially be more adequate. The sensor exhibits an accuracy of 0.25% over its full scale, corresponding to 0.025 PSI, and features an analog output, operating with a supply voltage of 3.3 volts [24]. While a narrower pressure range and higher accuracy would have been preferred, this sensor was also chosen due to its commercial availability.

**Figure 5:**
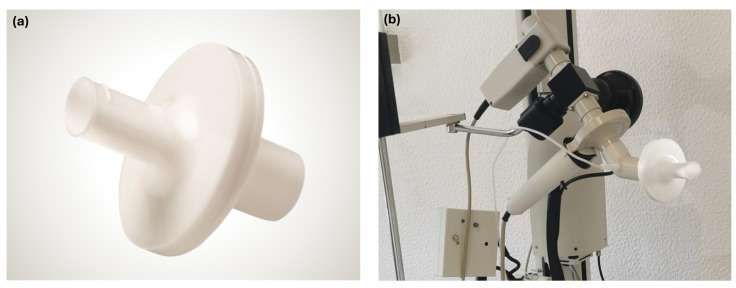
MicroGard^®^ IIB bacterial-viral filter (Vyaire Medical GmbH, Hoechberg, Germany). (**a**) MicroGard^®^ IIB bacterial-viral filter with integrated oval mouthpiece. (**b**) MicroGard^®^ IIB bacterial-viral filter in use with the Master Screen Body/Diffusion Sentry Suite at Hospital Pulido Valente.

In flow-sensing spirometers like the one proposed here, all exhaled air from the patient passes through the spirometer before being released into the room, potentially spreading contaminated droplets and aerosolized microorganisms [3]. To mitigate the risk of cross-infection and environmental contamination, a mouthpiece with a bacterial-viral filter, such as the MicroGard IIB (Vyaire Medical GmbH, Hoechberg, Germany), is placed between the patient’s mouth and the spirometer [3,26]. The MicroGard IIB filter, shown in Figure 5a, provides exceptional filtration efficiency, with a 99.999% effectiveness in preventing viral and bacterial cross-contamination [26]. This filter was chosen for its well-established use at Hospital Pulido Valente, as shown in Figure 5b, where clinical prototype testing took place. However, with an external diameter of 34 mm, modifications to the original Venturi tube design were required to accommodate its dimensions, as previously mentioned.

**Figure 6:**
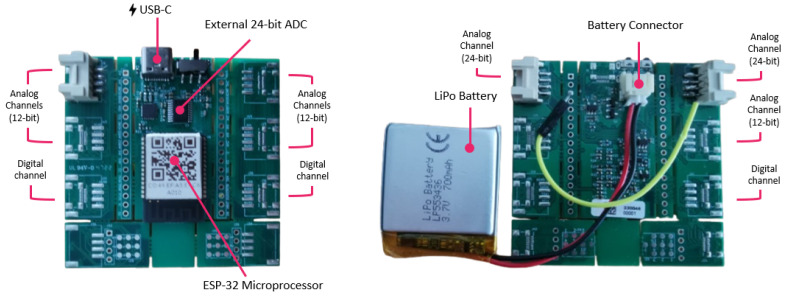
Overview of the ScientISST Sense acquisition board.

Finally, a circuit board is necessary for acquisition and wireless streaming of pressure sensor data. For this purpose, the ScientISST Sense acquisition board, developed at Instituto de Telecomunicações (IT) and depicted in Figure 6, was employed [27]. Featuring an ESP32 microcontroller, this board supports 12-bit analog-to-digital conversion and various wireless communication protocols (Wi-Fi, Bluetooth, and Bluetooth Low Energy (BLE)) [28]. Moreover, it operates with lithium-ion polymer batteries with a minimum supply voltage of 3.3 V, and it has a USB-C connector for battery recharging and serial communication with a computer.

In compliance with the American Thoracic Society (ATS)/ERS statement, which specifies a minimum sampling rate of ≥100 Hz and a resolution of at least 12 bits for digitizing the flow or volume signal [3], the sampling frequency was set to 1 kHz. The latest version of the ScientISST Sense acquisition board, equipped with a 24-bit external analog-to-digital converter (ADC), was used, which resulted in a resolution of 6.56 × 10^−5^ cm H_2_O.

**Figure 7:**
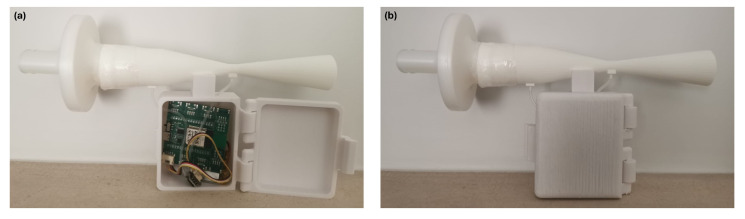
Prototype of the Venturi-based spirometer. (**a**) Prototype when the hinge box is opened, showing the ScientISST Sense acquisition board and the pressure sensor inside. (**b**) Prototype when the hinge box is closed—this configuration represents the intended usage during user testing.

Figure 7 shows a detailed view of the Venturi-based spirometer prototype. Aside from the previously described components, it also includes a box with a hinged lid. This box was specifically designed to house both the ScientISST acquisition board and the pressure sensor, increasing the prototype’s usability during clinical testing.

## 4. Computational Flow Dynamics

A fluid flow simulation was conducted on the Venturi tube using the advanced capabilities of SOLIDWORKS^®^ Flow Simulation 2022 (Dassault Systèmes), crucial for understanding how fluid behaves within the Venturi tube.

**Figure 8:**
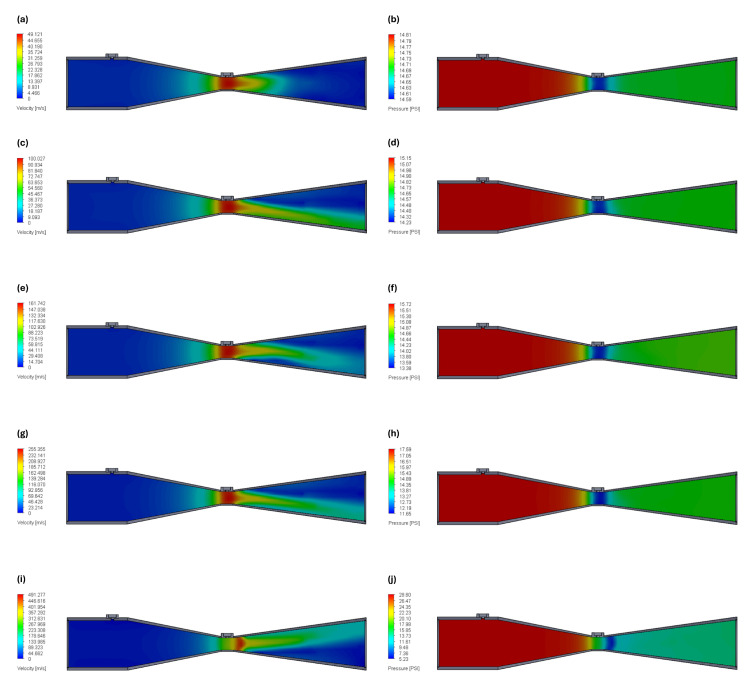
Velocity and pressure contours plots of the Venturi tube for different inlet velocities, created with SolidWorks^®^ Flow Simulation. (**a**) Velocity contour plot for boundary velocity of 3 m/s. (**b**) Pressure contour plot for boundary velocity of 3 m/s. (**c**) Velocity contour plot for boundary velocity of 6 m/s. (**d**) Pressure contour plot for boundary velocity of 6 m/s. (**e**) Velocity contour plot for boundary velocity of 9 m/s. (**f**) Pressure contour plot for boundary velocity of 9 m/s. (**g**) Velocity contour plot for boundary velocity of 12 m/s. (**h**) Pressure contour plot for boundary velocity of 12 m/s. (**i**) Velocity contour plot for boundary velocity of 15 m/s. (**j**) Pressure contour plot for boundary velocity of 15 m/s.

To analyze fluid flow in the Venturi tube, the model had to be fully closed with lids. The lid surfaces that come into contact with the fluid were used for applying boundary conditions. The outlet condition was set to environmental pressure (≈14.70 PSI), while the inlet condition was set to velocity. Flow simulations were conducted across inlet velocities ranging from 3 to 15 m/s, corresponding to flow rates of 2.26 L/s to 11.32 L/s. Results are depicted in Figure 8, showcasing velocity and pressure contour plots for each boundary velocity.

In Table 1, the numerical results of the simulations are presented; these include velocity, density, and pressure values for both the inlet and throat sections of the Venturi tube. The table also provides the calculated flow rates for these sections, derived from the respective simulated velocity values and cross-sectional areas.

The velocity contour plots clearly reveal the presence of flow separation at the diverging section for boundary velocities above 3 m/s. Although this phenomenon is not desirable, it appears to be confined to that specific region and, as a result, does not significantly impact the measurements. This would, however, have to be taken into account in bidirectional Venturi-based spirometers.

Moreover, it is also clearly illustrated that, except for a boundary velocity of 15 m/s, the maximum velocity consistently occurs at the Venturi tube’s throat. Likewise, the minimum pressure is consistently observed at the throat for all inlet velocities except for 15 m/s. To further understand why this expected behavior is not observed for a boundary velocity of 15 m/s, a detailed analysis of the data in Table 1 is required.

According to Table 1, the pressure difference between the inlet and the throat of the Venturi tube for a boundary velocity of 15 m/s is 12.45 PSI, which significantly deviates from the expected value. This discrepancy can be attributed to the exceptionally high velocity recorded at the throat, nearly reaching the speed of sound in air. Indeed, for velocities exceeding 100 m/s, air can no longer be treated as an incompressible fluid, leading to variations in air density across the tube [29]. As a result, the prototype cannot accurately measure flows exceeding 9 L/s, as these flows correspond to pressure differences above 5 PSI, which is beyond the sensor’s capacity. This limitation should be addressed in future iterations.

## 5. Laboratory Evaluation

### 5.1. Pressure Sensor Calibration

**Figure 9:**
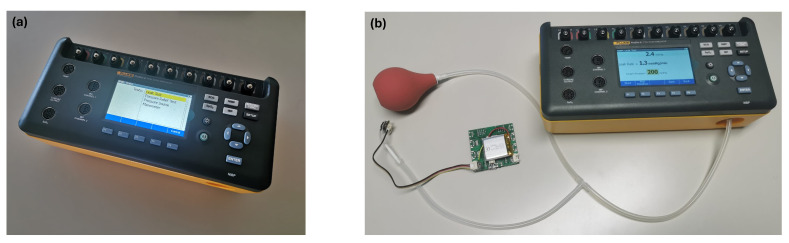
ProSim 8 Vital Signs Patient Simulator and setup for the calibration of the pressure sensor. (**a**) ProSim 8 Vital Signs Patient Simulator displaying the NIBP menu with four tests: leak test, pressure relief test, pressure source test, and manometer check. (**b**) Setup for the calibration of the pressure sensor, featuring the connection between the ProSim 8 pressure port, a rubber bulb, and the pressure sensor using silicone tubing.

A pressure sensor converts a pressure signal into an electrical signal output, which is only valuable if it accurately reflects the applied pressure. Therefore, the Honeywell SSCMRRN005PDAA3 pressure sensor underwent calibration against the ProSim 8 Vital Signs Patient Simulator (Fluke Biomedical, Everett, WA, USA) [30]. The ProSim 8 is a portable and versatile device capable of performing various preventative maintenance tests on patient monitors, including electrocardiogram (ECG), respiration, temperature, invasive blood pressure (IBP), cardiac output, non-invasive blood pressure (NIBP), oxygen saturation (SpO2), and rainbow multi-wavelength waveforms. To calibrate the pressure sensor, tests from the NIBP menu were selected and conducted, as illustrated in Figure 9a.

Firstly, the ProSim 8 pressure port was connected to both a pressure source (rubber bulb) and the pressure sensor. This connection was established using a silicone tube and a three-way silicone tube hose, as depicted in Figure 9b. Before proceeding with the calibration, an air leak test was conducted to ensure airtight connections and prevent any potential inaccuracies due to air leakage.

During the air leak test, the ProSim 8 pressurized the system to a target pressure of 200 mmHg (approximately 3.867 PSI). Pressure loss was continuously monitored on the Prosim 8 screen for two durations: 2 min and 4 min, each repeated 10 times. The average air leakage rate was 2.18 mmHg/min (standard deviation (SD) = 0.36) for the 2 min test and 1.52 mmHg/min (SD = 0.13) for the 4 min test. Converting to PSI, this corresponds to approximately 0.042 PSI (SD = 0.007) for the 2 min test and 0.029 PSI (SD = 0.007) for the 4 min test. These findings suggest that air leakage tends to stabilize or decrease over time during testing. Given that the mean air leakage rate was less than 1.5% of the target pressure, it was reasonable to conclude that the system has airtight connections and, therefore, to proceed with the calibration process as intended.

**Figure 10:**
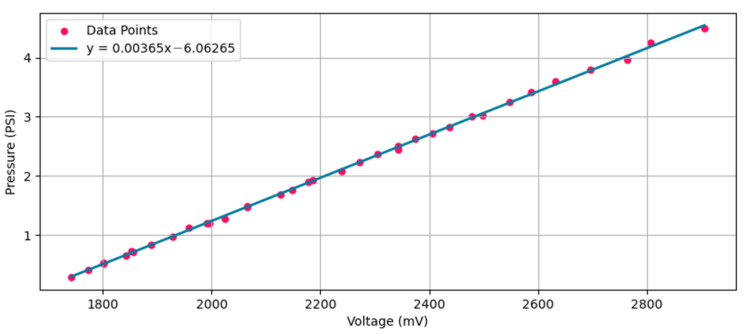
Calibration curve for the Honeywell SSCMRRN005PDAA3 pressure sensor, derived from 35 calibration data points (pink) and represented by the blue curve. The ProSim 8 served as the reference device.

The calibration process consisted of compressing a rubber bulb to generate pressure, which was simultaneously transmitted to both the ProSim 8 and the pressure sensor through silicone tubing. The peak pressure and the peak voltage were recorded from the ProSim 8 and the pressure sensor, respectively. This procedure was repeated 35 times, each time adjusting the degree of squeezing on the rubber bulb to create various pressure levels up to 4.5 PSI. A linear regression analysis was then performed to establish a precise relationship between the sensor’s voltage output and the applied pressure. Both the calibration data points and the calibration curve are presented in Figure 10. The calibration curve is expressed by Equation (5), with *y* representing the pressure measured in PSI and *x* corresponding to the sensor’s voltage output in millivolts. According to Equation (5), the sensitivity of the pressure sensor is 0.00365 PSI/millivolt.

(5) y=0.00365x−6.06265

### 5.2. Prototype Calibration Verification

**Figure 11:**
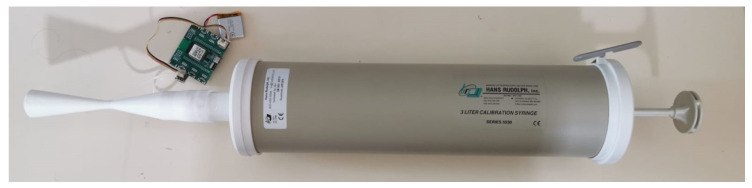
Setup for the calibration verification of the Venturi-based spirometer prototype with a 3 L calibration syringe (Hans Rudolph, KS, USA) [31].

To maintain spirometer accuracy and reliability, the Spirometry Technical Statement issued by the ATS and the ERS recommends daily calibration verification using a 3 L syringe with an accuracy of ±15 mL or ±0.5% of the full scale [3]. A spirometer must report a value of 3 L ± 3%; otherwise, recalibration is necessary [3]. Therefore, before clinical testing, the spirometer prototype underwent calibration verification with a 3 L calibration syringe (Hans Rudolph, KS, USA), which has an accuracy of ±15 mL as recommended, available at the Lisbon Biomechanics Laboratory (Lisbon, Portugal) [31]. Typically, if an in-line filter is used in spirometry testing, it should also be employed during recalibrations and verifications [3]. However, due to its oval shape, the calibration verification setup (shown in Figure 11) did not include the MicroGard IIB filter.

**Figure 12:**
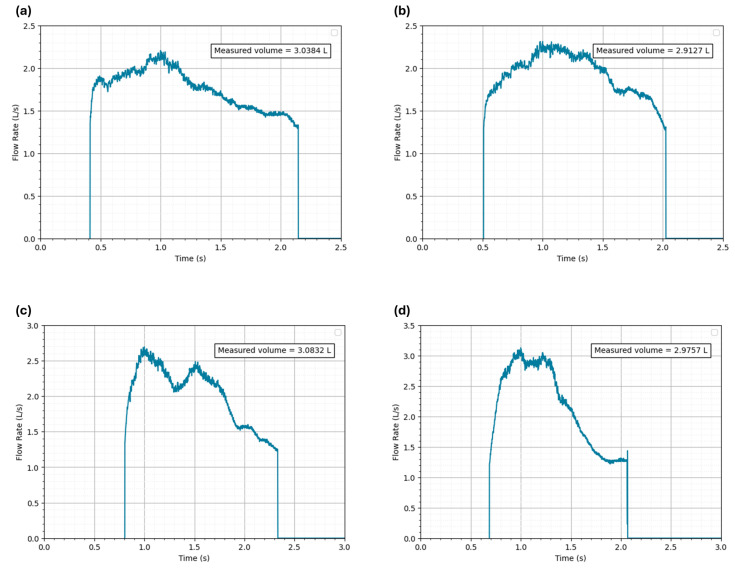
Flow rate-time curves of the calibration verification tests with a 3 L syringe, indicating: (**a**) measured volume of 3.0384 L; (**b**) measured volume of 2.9127 L; (**c**) measured volume of 3.0832 L; and (**d**) measured volume of 2.9757 L.

Four successive tests were performed. The volume was computed by integrating the measured flow, which was calculated using Equation (4). The respective flow rate-time curves are shown in Figure 12. The syringe did not have an automatic flow rate control, so the flow rate was not precisely controlled, and variations in flow rate did occur. It is also important to note that these results were obtained for a $C_{d}$ of 0.97.

The average measured volume was 3.0025 L, with a standard deviation of 0.0743 L. All measured volumes were within the limits established by the ATS/ERS standards. The variability observed in the results can be attributed to the varying speeds at which the syringe was emptied into the Venturi tube for each test.

## 6. Experimental Evaluation

The spirometer prototype underwent evaluation with human subjects to capture the natural variability of the body. This included testing its test-retest reliability through repeated measures and assessing concurrent validity by comparing its results with those obtained from a gold-standard spirometer. Due to the suitable technical conditions, this study was carried out at the Respiratory Pathophysiology Laboratory of Hospital Pulido Valente (Lisbon, Portugal), under the supervision of a trained technician.

### 6.1. Materials and Methods

#### 6.1.1. Participants

Eight individuals participated in this study. The inclusion criteria were individuals aged 18 years and older, with no respiratory conditions or contraindications to the tests. The purpose and procedure of the study were explained to all participants.

#### 6.1.2. Equipment

The user tests employed two different devices: a hospital-grade plethysmograph and the Venturi-based spirometer prototype. The goal was to compare the performance of the prototype with the Pulmonary Function Test (PFT) device by measuring both the Forced Vital Capacity (FVC) and Forced Expiratory Volume in one second (FEV_1_), the most clinically relevant parameters of lung function derived from spirometry [16].

**Figure 13:**
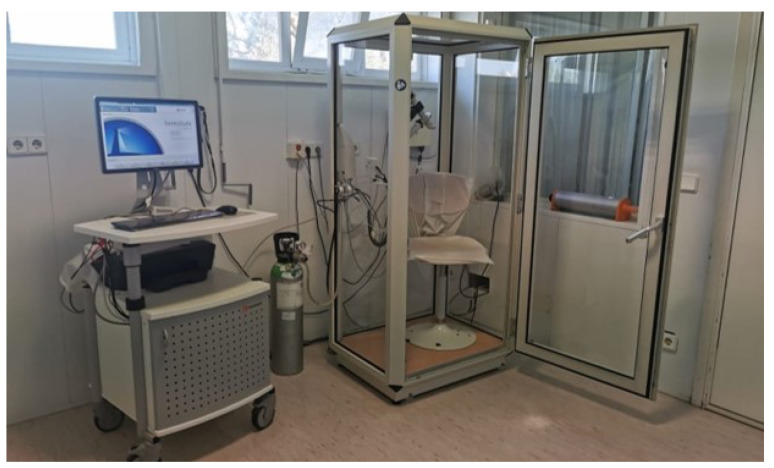
MasterScreen Body/Diffusion (Carefusion, Hoechberg, Germany), a whole-body plethysmograph equipped with a pneumotachograph for spirometry.

The PFT equipment used was the MasterScreen Body/Diffusion (Carefusion, Hoechberg, Germany), a well-regarded whole-body plethysmograph capable of comprehensive pulmonary function testing. This advanced system, illustrated in Figure 13, includes a pneumotachograph for spirometry which can detect volumes up to 20 L. It operates with a margin of error of ±3% or ±0.05 L (whichever is greater) for volumes within the range of 0.5 to 8 L. The flow rate range is 0–20 L/s, with a margin of error of ±2% or ±0.2 L/s (whichever is greater) for flows between 0.2 and 12 L/s.

The prototype device is already shown in Figure 7. Unlike the hospital-grade plethysmograph, which requires a dedicated testing environment and technician assistance, this prototype empowers participants to conduct tests independently. In this study, pressure data were transmitted in real-time to a computer via Bluetooth, allowing for immediate calculation of spirometric parameters. However, in future iterations, this calculation could be performed directly by a mobile application, offering users greater convenience and flexibility.

#### 6.1.3. Protocol

Each participant performed spirometry on the plethysmograph, followed by a 2 min pause before testing with the prototype. All tests adhered strictly to ATS/ERS guidelines, requiring participants to execute at least three acceptable spirometry maneuvers on each device. Additional tests were conducted if necessary to meet repeatability criteria.

As the prototype was designed for expiration-only maneuvers, the spirometry test procedure had to be adapted accordingly. Initially, the patient executes a rapid inspiration to Total Lung Capacity (TLC). Within 2 s, they insert the mouthpiece, form a seal with their lips, and initiate maximal expiration [3]. To minimize air loss before sealing the lips, it was suggested to keep the mouth open until the mouthpiece is securely positioned [3]. For the hospital-grade device, standard procedure was followed.

**Figure 14:**
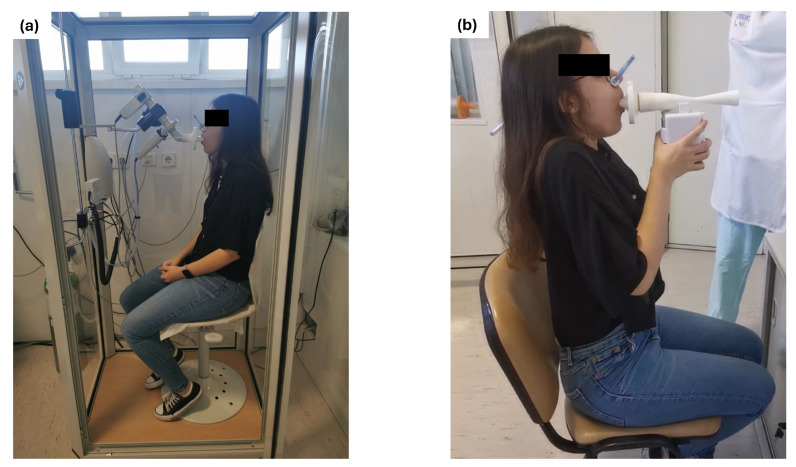
A participant performing spirometry tests during the clinical study: (**a**) in the plethysmograph; (**b**) with the Venturi-based prototype.

Figure 14 shows a participant undergoing spirometry tests using both the plethysmograph and the prototype. All tests were performed with participants seated and using a mouthpiece with an integrated bacterial filter, along with a nose clip to ensure that air flows exclusively through the mouth.

#### 6.1.4. Statistical Analysis

Statistical analysis was performed using the SPSS software (IBM SPSS Statistics 29.0, Armonk, New York, NY, USA). The normality of the lung function variables was assessed using the Shapiro–Wilk test, suitable for small sample sizes (fewer than 50 samples). Differences between the values measured by the two devices were analyzed using a paired *t*-test, which assumes normally distributed data.

Concurrent validity between the prototype and the hospital-grade device was evaluated through the Pearson correlation coefficient (*r*), which measures the strength of linear association between variables. Concurrent validity was also assessed using the intraclass correlation coefficient (ICC), a measure used to determine if subjects can be consistently rated by different raters, such as technicians, devices, laboratories, or procedures. In this study, each participant underwent assessment with two different devices. Therefore, the two-way random effects model, ICC(2,1), was employed, which assumes that both raters and subjects contribute to random effects, thereby accounting for measurement error. ICC values range from 0 to 1, indicating reliability levels as follows: poor reliability (ICC <0.50), moderate reliability (0.50≤ ICC <0.75), good reliability (0.75≤ ICC <0.90), or excellent reliability (ICC ≥0.90).

The test-retest reliability was also assessed using the ICC. For this analysis, the ICC coefficient was computed from the three acceptable and repeatable spirometric maneuvers employing a two-way mixed effects model, denoted as ICC(3,1). In this model, systematic errors are not considered as measurement error because the rater (device) remains consistent across the three measurements.

Finally, the level of agreement between the two devices was assessed via a Bland–Altman analysis, the preferred method for evaluating agreement between medical instruments that measure continuous variables [32]. This analysis involved creating a scatter plot where the differences between paired measurements were plotted against their averages. Horizontal lines were drawn at the mean difference (bias) and at the 95% limits of agreement (mean difference ±1.96 SD of the differences), which quantify the accuracy and precision of the new device, respectively. An underlying assumption of this method is that the differences between the paired measurements are normally distributed.

A *p*-value of <0.05 was considered statistically significant, and a *p*-value of <0.001 was considered highly statistically significant.

## 7. Results

All eight recruited participants met the inclusion criteria and were included in the study. Table 2 displays their anthropometric characteristics and lung function measures obtained with the hospital-grade device.

The Shapiro–Wilk test results suggested no evidence to reject the assumption of normal distribution for the differences between measurements obtained with the two devices. Specifically, the *p*-values for FVC and FEV_1_ were 0.317 and 0.641, respectively.

Subsequently, the results of the paired *t*-test are presented in Table 3. This analysis showed no statistically significant difference between the prototype and the hospital-grade device for both FVC and FEV_1_ (*p*-value > 0.05).

The results of the test-retest reliability evaluation are presented in Table 4. Notably, FEV_1_ demonstrated good reliability (ICC > 0.8), while FVC exhibited excellent reliability (ICC > 0.9).

Table 5 presents the Pearson correlation coefficient (*r*) and ICC for the prototype versus the plethysmograph. The *r* values show a strong, highly statistically significant correlation for both FVC and FEV_1_. Similarly, the ICC values indicate excellent reliability, also highly statistically significant, for both parameters.

**Figure 15:**
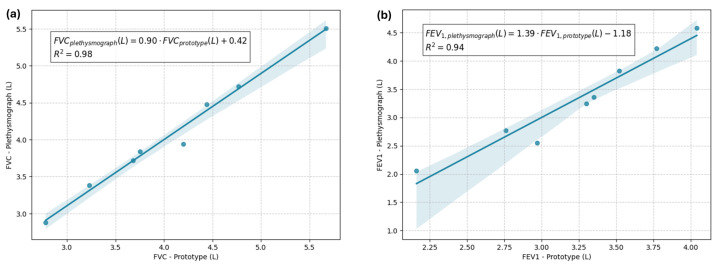
Scatter plots with regression lines comparing measurements from the prototype and the plethysmograph. (**a**) FVC measurements from both devices. (**b**) FEV_1_ measurements from both devices. Each plot includes a fitted regression line to illustrate the linear relationship between measurements from the two devices.

To visually assess the linear association between measurements obtained with the prototype and the plethysmograph, scatter plots were generated. Figure 15a illustrates the relationship between FVC measurements from both devices, while Figure 15b shows the relationship for FEV_1_. These plots confirm a strong linear association, consistent with the statistical findings.

**Figure 16:**
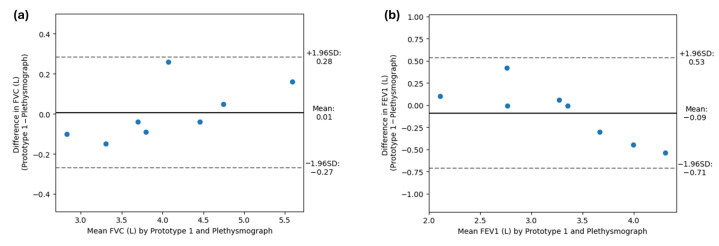
Bland−Altman plots. (**a**) Bland−Altman plot for the FVC. (**b**) Bland−Altman plot for the FEV_1_. The solid line indicates the mean difference (bias) between paired measurements, and the dotted lines indicate the 95% limits of agreement.

As the assumptions of normality were not previously rejected by the Shapiro–Wilk test, a Bland–Altman analysis was conducted, which revealed a bias close to zero for both FVC (0.0063 L) and FEV_1_ (−0.0912 L). These results, as well as the 95% limits of agreement, are summarized in Table 6. Bland–Altman plots for FVC and FEV_1_ are shown in Figure 16a and Figure 16b, respectively. Notably, all individuals were within the limits of agreement.

## 8. Conclusions

This paper presents the development and evaluation of a novel spirometer prototype based on the Venturi effect. The main contribution of this work is the design of a spirometer without moving parts and complex resistive elements, resulting in a simpler, more robust, and easily manufacturable device. Despite its straightforward design, the spirometer provides reliable measurements of the most clinically relevant lung function metrics derived from spirometry (FVC and FEV_1_). Furthermore, its user-friendly design allows patients to use the device independently, making it well-suited for home use. Additionally, this spirometer offers a cost-effective solution for flow measurement in spirometry, making it more affordable than most commercially available handheld spirometers, which often exceed EUR 1000 in cost.

It is important to note that the prototype measures airflow in only one direction. Despite this unidirectional design, it effectively measures FVC and FEV_1_. Future work could explore a bidirectional design by incorporating additional pressure sensors to assess all lung function parameters more comprehensively. Nonetheless, the simpler unidirectional design may be particularly well-suited for home monitoring, where affordability and ease of use are critical factors.

The prototype was tested with real users in a hospital setting under the supervision of a trained technician. Participants conducted the tests with the prototype themselves, reflecting the intended use of this portable device. In contrast, tests with the gold standard PFT equipment were conducted by a technician. The study demonstrated excellent test-retest reliability, with an ICC of 0.955 for FVC and 0.853 for FEV_1_. Additionally, comparisons with the gold standard PFT equipment revealed strong correlation (Pearson correlation coefficient of 0.992 for FVC and 0.968 for FEV_1_) and high reliability (ICC of 0.987 for FVC and 0.907 for FEV_1_). The Bland–Altman analysis showed minimal bias and narrow limits of agreement, with a bias of 0.006 L for FVC (limits of agreement: −0.270 L to 0.283 L) and a bias of −0.091 L for FEV_1_ (limits of agreement: −0.714 L to 0.532 L). These results confirm the prototype’s accuracy and precision, and are comparable to those reported in the literature for portable spirometers.

Overall, the Venturi-based spirometer demonstrates significant potential as a cost-effective alternative to commercially available handheld spirometers. By providing a reliable and affordable option, this prototype can significantly enhance access to spirometry, enabling more individuals to monitor their respiratory health conveniently and effectively in the comfort of their own homes. This advancement holds promise for improving early detection and management of respiratory conditions, ultimately contributing to better health outcomes and quality of life.

## 9. Future Work

To advance the Venturi-based spirometer prototype, several key improvements can be implemented. These include conducting a thorough analysis of how different Venturi tube diameters affect airflow measurements to enhance accuracy and optimize device portability, as well as enhancing the airtightness of the connection between the Venturi tube and the bacterial filter. Additionally, exploring alternative materials or manufacturing techniques could mitigate issues such as micro-holes commonly observed in 3D-printed parts made with PLA, ensuring consistent performance and reliability.

Furthermore, to ensure the spirometer’s reliability and durability over extended periods, rigorous testing is essential. Specifically, accelerated life testing will be conducted to mimic long-term usage and identify potential failure points. Additionally, cyclic stress testing will simulate frequent-use scenarios to evaluate the device’s reliability under continuous operation, ensuring it maintains consistent performance over time [33].

Integrating a smartphone app for data acquisition and analysis is another key objective, as it would provide real-time feedback on spirometry maneuvers and assess the quality of participant effort. Ensuring adherence to ATS/ERS guidelines for spirometry maneuvers is critical. This app would help guarantee that maneuvers meet these standards, thereby enhancing the accuracy and reliability of spirometry measurements.

Regarding the clinical evaluation, it is important to address some limitations identified in the study. Firstly, the sample size was small, involving only eight individuals. This limited sample size prevented the randomization of the device order during testing. Moreover, the Pearson correlation coefficient has been shown to overestimate reliability for small sample sizes. Nonetheless, the ICC, which does not overestimate relationships for small samples, was consistently high. Additionally, the study participants were all healthy individuals without a history of respiratory diseases, which limits the generalizability of the findings to populations with respiratory conditions.

As a result, future work should also focus on large-scale concurrent validation and test-retest reliability studies to further evaluate the prototype’s performance. These studies should include a diverse sample of participants, representing both healthy individuals and those with various respiratory diseases, to prevent potential biases and ensure reproducibility of results. Expanding the evaluation to include other spirometric parameters beyond FVC and FEV_1_ will also provide a more comprehensive assessment of the prototype’s capabilities.

## Figures and Tables

**Table 1:** Flow simulation results for the Venturi tube. The numerical values for velocity, density, pressure, and flow rate of the air flowing through position 1 (inlet) and position 2 (throat) of the Venturi tube for each boundary velocity ($v_{b}$) are displayed.

$v_{b}$ (m/s)	$v_{1}$ (m/s)	$v_{2}$ (m/s)	$\rho_{1}$ (kg/m^3^)	$\rho_{2}$ (kg/m^3^)	$\mu_{2}$ (Pa s)	$p_{1}$ (PSI)	$p_{2}$ (PSI)	$q_{v,1}$ (L/s)	$q_{v,2}$ (L/s)
3.00	3.05	48.20	1.21	1.20	1.81×10^−5^	14.81	14.60	2.30	2.42
6.00	6.07	98.80	1.24	1.19	1.79×10^−5^	15.15	14.29	4.58	4.97
9.00	9.10	158.83	1.29	1.16	1.75×10^−5^	15.72	13.50	6.87	7.98
12.00	12.08	249.34	1.44	1.10	1.65×10^−5^	17.59	11.96	9.12	12.53
15.00	15.41	304.91	2.31	1.46	1.66×10^−5^	28.52	16.07	11.63	15.33

**Table 2:** Anthropometric characteristics and lung function measures of the participants. Data are presented as mean ± SD, except for sex, which is presented as the number of males (*M*) and females (*W*).

Characteristic	Study Sample (N = 8)
Sex	3 M/5 W
Age (y)	37.75 ± 12.28
Height (cm)	168.12 ± 11.29
Weight (kg)	70.75 ± 17.60
BMI (kg/m^2^)	24.86 ± 5.06
FVC (L)	4.06 ± 0.82
FEV_1_ (L)	3.32 ± 0.86

Definition of abbreviation: BMI = Body Mass Index.

**Table 3:** Paired *t*-test results of the paired differences in the measurements of FVC and FEV_1_ between the prototype and the plethysmograph, as well their respective 95% confidence interval (CI) and *p*-values.

	Paired Differences		95% CI of the Difference		Significance
Metric	Mean	SD	Lower Bound	Upper Bound	*p*-Value
FVC (L)	0.0063	0.1410	−0.1116	0.1241	0.9040
FEV_1_ (L)	−0.0912	0.3180	−0.3571	0.1746	0.4440

**Table 4:** Intraclass correlation coefficients for the three repeated measurements of FVC and FEV_1_, as well as their respective 95% CI and *p*-values. The two-way mixed effects model (3,1) was employed.

Metric	ICC (3,1) (95% CI)	*p*-Value
FVC	0.955 (0.859 to 0.990)	<0.001
FEV_1_	0.853 (0.597 to 0.965)	<0.001

**Table 5:** Pearson correlation and intraclass correlation coefficients for the measurements of FVC and FEV_1_ between the prototype and the plethysmograph, as well as their respective *p*-values. The 95% CIs for ICC are also presented. The two-way random effects model (2,1) was employed.

	Pearson Correlation		Intraclass Correlation	
Metric	r	*p*-Value	ICC (2,1) (95% CI)	*p*-Value
FVC	0.992	<0.001	0.987 (0.936 to 0.997)	<0.001
FEV_1_	0.968	<0.001	0.907 (0.609 to 0.981)	<0.001

**Table 6:** Bias and 95% limits of agreement for the repeated measurements of FVC and FEV_1_ between the prototype and the plethysmograph, as well as the respective 95% CI.

Metric	Bias (95% CI)	Lower Limit of Agreement (95% CI)	Upper Limit of Agreement (95% CI)
FVC (L)	0.0063 (−0.1116 to 0.1241)	−0.2701 (−0.3880 to −0.1522)	0.2826 (0.1647 to 0.4005)
FEV_1_ (L)	−0.0912 (−0.3571 to 0.1746)	−0.7144 (−0.9803 to 0.4486)	0.5319 (0.2661 to 0.7978)

**Figure A1:**
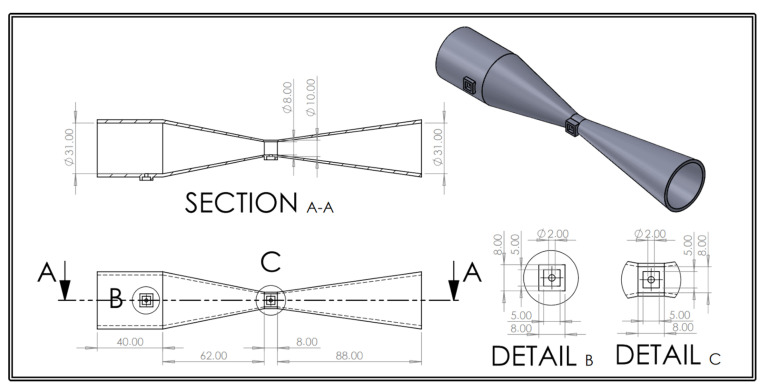
Technical drawing of the Venturi tube; all dimensions are presented in millimeters. Detail B and Detail C are proportionally scaled at a 1:2 ratio relative to the main drawing.

## Data Availability

The data presented in this study are available upon reasonable request made to the corresponding author. The data are not publicly available due to privacy restrictions.

## Abbreviations

The following abbreviations are used in this manuscript:

ADC	Analog-to-Digital Converter
ATS	American Thoracic Society
BLE	Bluetooth Low Energy
CAD	Computer-Aided Design
CI	Confidence Interval
ECG	Electrocardiogram
ERS	European Respiratory Society
FEV_1_	Forced Expiratory Volume in one second
FVC	Forced Vital Capacity
IBP	Invasive Blood Pressure
ICC	Intraclass Correlation Coefficient
ICCs	Intraclass Correlation Coefficients
IT	Instituto de Telecomunicações
NIBP	Non-Invasive Blood Pressure
PFT	Pulmonary Function Test
PLA	Polylactic Acid
PSI	Pounds per Square Inch
SD	Standard Deviation
SpO_2_	Oxygen Saturation
SPSS	Statistical Package for Social Sciences
TLC	Total Lung Capacity

## Appendix A. CAD Design and Manufacturing of the Venturi Tube

### Appendix A.1. Initial CAD Design

The Venturi tube was designed using the CAD software SolidWorks 2022 (Dassault Systèmes). The technical drawing of the initial design is provided in Figure A1.

### Appendix A.2. Revised CAD Design

Design revisions were necessary due to practical constraints: the bacterial filter used has an external diameter of 34 mm, and the 3 L calibration syringe has an external diameter of 35 mm. The revised design is presented in Figure 3 of the main text.

### Appendix A.3. Pressure Tap Design

The Venturi tube features two openings intended for pressure taps. These taps were designed as barbed hose fittings, with ridges and bumps, to grip the inside diameter of the silicone tube and seal the connection. The technical drawing of these pressure taps is shown in Figure 4 of the main text.

### Appendix A.4. 3D-Printing and Assembly

The Venturi tube and pressure taps were manufactured in a Creality Ender 3 Pro 3D printer (Shenzhen Creality 3D Technology, Shenzhen, China) [34]. To convert the exported SOLIDWORKS^®^ STL files into the G-code format, the Ultimaker Cura slicing software (Ultimaker BV, Utrecht, The Netherlands) was employed [35]. White polylactic acid (PLA) filament with a diameter of 1.75 mm was the chosen material for the printing process. After printing, the pressure taps were inserted into the Venturi tube and superglued in place.
